# Barriers and preferences in advance care planning: a mixed-methods study of end-stage kidney disease patients and caregivers in India

**DOI:** 10.1186/s12882-025-04354-2

**Published:** 2025-07-24

**Authors:** Bharathi Naik, Anuja Damani, Arun Ghoshal, Shankar Prasad Nagaraju, Pankaj Singhai, Swathi Nayak Ammunje, Ravindra Prabhu Attur, Naveen Salins

**Affiliations:** 1 Department of Renal Replacement Therapy and Dialysis Technology, Manipal College of Health Professions Manipal, Manipal Academy of Higher Education, Manipal, Karnataka 576 104 India; 2 Department of Palliative Medicine and Supportive Care, Kasturba Medical College Manipal, Manipal Academy of Higher Education, Manipal, Karnataka 576 104 India; 3 Department of Nephrology, Kasturba Medical College Manipal, Manipal Academy of Higher Education, Manipal, Karnataka 576 104 India; 4 Department of Palliative Medicine, Sri Aurobindo Medical College and PG Institute, Sri Aurobindo University, Indore, 453555 India

**Keywords:** Advance care planning, End-stage kidney disease, Dialysis, India, Patient-centered care, Family involvement

## Abstract

**Objectives:**

To understand preferences for illness-related information, decision-making roles, and barriers to Advance Care Planning (ACP) documentation among patients with End-stage Kidney Disease (ESKD) and their caregivers in India.

**Methods:**

A convergent parallel mixed-methods study was conducted from October 2022 to September 2023 at a tertiary care hospital in South India. Quantitative data were collected using a validated ACP questionnaire from 247 patient-caregiver dyads. Qualitative data were obtained from semi-structured interviews with 34 patients and 6 caregivers. Quantitative data were analyzed using descriptive and inferential statistics; qualitative data were analyzed thematically using Braun and Clarke’s framework.

**Results:**

Most patients (90.7%) and caregivers (94.1%) preferred detailed illness-related information, yet ACP documentation interest was low (20.4% dialysis, 9.2% non-dialysis patients). The majority preferred collaborative decision-making involving family and clinicians. Barriers to ACP included limited awareness, emotional burden, and systemic challenges. Enablers included family support and provider guidance.

**Conclusions:**

While interest in illness information and shared decision-making is high, ACP documentation remains limited. Tailored strategies are needed to promote ACP engagement.

**Practice implications:**

Integrating culturally sensitive ACP discussions into routine nephrology care and involving families can improve ACP participation.

**Supplementary Information:**

The online version contains supplementary material available at 10.1186/s12882-025-04354-2.

## Introduction

End-stage kidney disease (ESKD) represents the final stage of chronic kidney dysfunction and is associated with significant treatment decisions and quality-of-life considerations. Patients with ESKD require either renal replacement therapy (dialysis or transplant) or may opt for conservative management [1]. These treatment pathways often raise complex issues that necessitate timely and inclusive communication regarding care goals and patient preferences.

Advance Care Planning (ACP) is a process that facilitates discussions about future healthcare preferences, including the designation of surrogate decision-makers and documentation of choices regarding treatment and care [2]. ACP is widely recognized as essential to patient-centered care for individuals with serious illnesses, including ESKD [3]. However, in India, ACP remains underutilized due to several factors: lack of structured protocols, limited awareness among patients and families, cultural sensitivities around death and prognosis, and systemic challenges such as inadequate training of healthcare providers and time constraints in clinical practice [4–8].

In India’s collectivist society, decision-making in healthcare is predominantly family-centered. Cultural taboos around discussing end-of-life care, combined with reluctance to talk about mortality, often delay or prevent ACP conversations. Additionally, physicians may feel unequipped or unsupported in facilitating these discussions due to a lack of training or guidance. Prior studies have emphasized the importance of context-specific approaches to ACP, yet research in Indian nephrology settings remains sparse [9–12].

This study aims to explore how ACP can better support the care of Indian patients with ESKD by examining preferences and barriers from both patient and caregiver perspectives. Using a convergent parallel mixed-methods approach, we investigate how patients and caregivers perceive illness-related information, make decisions regarding treatment, and engage (or choose not to engage) in ACP documentation. The study seeks to offer an understanding of culturally relevant practices and inform strategies for integrating ACP into routine nephrology care in India.

## Methods

### Study design and setting

This study employed a convergent parallel mixed-methods design [13], conducted between October 2022 and September 2023 at Kasturba Hospital, a tertiary care teaching hospital in Manipal, India. The mixed-methods approach allowed for the simultaneous collection and analysis of quantitative and qualitative data, facilitating triangulation and a comprehensive understanding of ACP perspectives.

### Participants

Participants included adults (≥ 18 years) diagnosed with End-stage Kidney Disease (ESKD) and their primary caregivers. ESKD was defined as kidney failure requiring dialysis or being managed conservatively, based on clinical diagnosis and estimated glomerular filtration rate (eGFR < 15 mL/min/1.73 m²) [1]. Exclusion criteria for patients included acute kidney injury, inability to comprehend the study due to cognitive impairment or language barrier, or those too unwell to participate. Caregivers were defined as individuals involved in the patient’s care for at least six months. Patient-caregiver dyads were enrolled, and participation was not permitted independently by either group.

### Sampling and recruitment

A total of 247 patient-caregiver dyads were recruited using convenience sampling. All eligible patients and their matched caregivers were approached during nephrology outpatient or dialysis unit visits. Patients who choose to forego dialysis (non – dialysis patients) were managed under conservative kidney management programs led by the nephrology team, which involve symptom control, fluid management, and psychosocial support [14]. Written informed consent was obtained from both patients and caregivers in their preferred language.

### Quantitative data collection

A previously validated questionnaire on Advance Care Planning (ACP) preferences was administered by trained research assistants fluent in local languages. The questionnaire had separate but parallel forms for patients and caregivers, each containing 16 items across two domains: (1) knowledge and understanding of illness and care needs, and (2) preferences for healthcare decision-making and ACP documentation. This tool had been validated in a prior pilot study with Cronbach’s alpha scores of 0.925 (patients) and 0.875 (caregivers) [5].

### Qualitative data collection

Semi-structured interviews were conducted with a purposive sample of 34 patients and 6 caregivers selected to represent diverse age, gender, dialysis status, and caregiving roles. Interviewees were selected until data saturation was reached, defined as the point where no new themes emerged [15]. Interviews were conducted separately for patients and caregivers, in private spaces within the hospital, by experienced qualitative researchers (BN and AG). An interview guide, informed by literature and expert input, was used. Interviews lasted 20–30 min, were audio-recorded, transcribed verbatim, and translated to English where needed.

### Data analysis

**Fig. 1:**
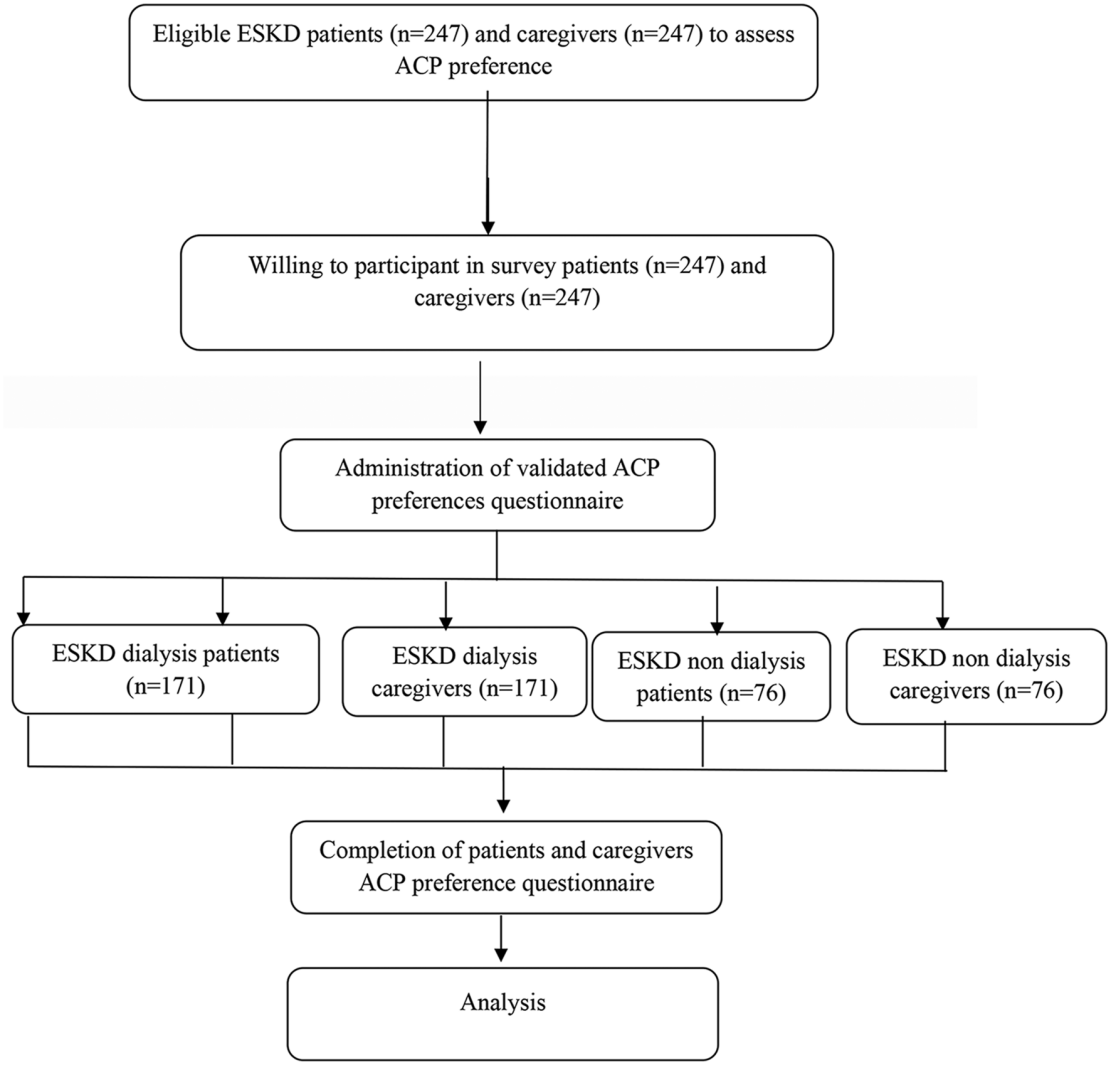
Flow diagram of the study

Quantitative data were analyzed using SPSS Version 21. Descriptive statistics (means, SDs, frequencies) were used to summarize responses. The chi-square test was used to compare dialysis and non-dialysis subgroups, and p values less than 0.05 were considered statistically significant [16]. Responses on Likert scales were dichotomized for interpretability (e.g., “never” as No; other responses as Yes). Qualitative data were analyzed using Braun and Clarke’s thematic analysis framework [17]. An inductive approach was used, meaning that themes were generated from the data itself rather than pre-set codes. Two researchers (BN and AG) independently coded transcripts and resolved discrepancies through discussion. Themes were reviewed iteratively and summarized across participants (please see Fig. 1).

### Ethical considerations

This study was conducted in accordance with the ethical principles outlined in the Declaration of Helsinki, and it was approved by the Institutional Review Board (or Ethics Committee) of the Kasturba Medical College, Manipal (IEC-214/2021). Written informed consent was obtained from all participants prior to enrollment.

## Results

### Demographic characteristics

The average age of ESKD patients was 51.9 ± 14.5 years, and caregivers had a mean age of 42.8 ± 12.8 years. Most patients (76.9%) were male, while most caregivers (64.4%) were female. A significant proportion of participants (59.5%) resided in rural areas. Higher education was reported in 24.3% of patients and 52.2% of caregivers. Most caregivers (98.4%) were first-degree relatives (please see Table 1).

**Table 1:** Demographic characteristics of ESKD patients and their caregivers *(A summary of age*,* gender*,* education*,* marital status*,* region*,* relation to the patient*,* and income distribution among Dialysis and non-dialysis groups)*

Demographic variables	ESKD patients on Dialysis − 171 (No. %)	ESKD patients not on dialysis-76 (No. %)	Total ESKD patients − 247 (No. %)
**Age (mean ± SD)**	51.9 ± 14.0	52.1 ± 15.6	51.9 ± 14.5
**Gender**			
Male	134(54.25)	56(22.67)	190(76.9)
Female	37(14.97)	20(8.09)	57(23.1)
**Region**			
Rural	100(40.48)	47(19.02)	147(59.5)
Urban	71(28.7)	29(11.74)	100(40.5)
**Education Status**			
No Education	11(4.45)	5(2.02)	16(6.5)
Primary Education	54(21.86)	29(11.75)	83(33.6)
Secondary Education	60(24.29)	28(11.34)	88(35.6)
Higher Education	46(18.62)	14(5.66)	60(24.3)
**Marital Status**			
Married	133(53.84)	61(24.69)	194(78.5)
Single	31(12.55)	9(3.64)	40(16.2)
Widowed	7(2.83)	6(2.429)	13 (5.3)
**Religion**			
Hindu	145(58.70)	72(29.14)	217(87.9)
Muslim	14(5.66)	2(0.8)	16(6.5)
Christian	12(4.85)	2(0.8)	14(5.7)
**Types of Family**			
Joint	45(18.21)	27(10.93)	72(29.1)
Nuclear	126(51.02)	49(19.84)	175(70.9)
**Relation with patients**			
First-degree relatives, including spouses	166(67.2)	69(27.93)	235(95.14)
Second-degree relatives (cousins, nephews)	5(2.02)	3(1.21)	8(3.24)
Friends	0(0%)	4(1.61)	4(1.61)
**Financial Status (monthly income of family in INR)**			
< 50 k	143(57.89)	54(21.87)	197(79.75)
50–100 k	3(1.22)	2(0.81)	5(2)
> 100k	16(6.48)	7(2.84)	23(9.3)
NA	9(3.65)	13(5.27)	22(8.9)

Table 2 presents the responses of patients with End-Stage Kidney Disease (ESKD) regarding their knowledge and experiences related to kidney palliative care (referring to comprehensive, multidisciplinary symptom and psychosocial support for patients with ESKD). The responses are stratified by dialysis status, comparing patients on dialysis (*n* = 171) and those managed conservatively without dialysis (*n* = 76). None of the dialysis patients and only one non-dialysis patient reported having received palliative care for symptom management, and even those who were somewhat familiar with it showed minimal recognition of its scope and benefits. A small number of patients acknowledged supportive measures for managing symptoms, but most expressed limited or no awareness; among those aware, responses about non-pharmacological interventions varied significantly. Moreover, very few patients could articulate what palliative or supportive care (general comfort measures and non-curative medical interventions) entails, with many responding with “no idea” or leaving the question blank, highlighting a substantial knowledge gap. Interestingly, when asked hypothetically about considering supportive care if recommended by a physician, a major portion of both groups expressed openness to it, demonstrating a willingness to explore supportive care despite their limited initial awareness. Overall, these findings underscore a significant lack of exposure to and understanding of kidney palliative care among ESKD patients, which may serve as a critical barrier to Advance Care Planning (ACP) if patients remain unaware that options for symptom relief, quality-of-life support, and care planning are part of palliative care.

**Table 2:** Knowledge of kidney palliative care among patients with ESKD (Comparison of awareness, understanding, and willingness to consider palliative care between Dialysis and non-dialysis patients)

Knowledge questions regarding palliative care in ESKD patients	ESKD patients on dialysis (*n* = 171)		ESKD patients not on dialysis(*n* = 76)	
	Yes	No	Yes	No
Whether received palliative care for any symptoms • If yes, which symptom, • Has it improved quality of life	0(0%)	171(100%)	1(1.3%) (Treatment for Sleep disorder improved this symptom)	75(98.7%)
Whether the patient knows symptoms can be treated by “supportive measures.” • If Yes- what measures • If No- are they interested in receiving supportive care If doctors suggested Not decided Yes, if necessary	1(0.6%)	170(99.4%)	1(1.3%)	75(98.7%)
	Nonpharmacological		Nonpharmacological	
		7(4.1%) 111(64.9%) 53(31.0%)		2(2.6%) 41(53.9%) 33(43.4%)
What do they think palliative care is/ idea of supportive care? • If Yes- They think it was good • If No- no idea about supportive care	16(9.4%)	155(90.6%)	2(2.6%)	74(97.4%)

### Preferences for illness information

Among dialysis patients, 90.7% wished to be informed about their illness, and 79.5% about its severity. Similarly, 94.1% of caregivers desired detailed illness information. Non-dialysis patients and caregivers showed slightly lower preferences (patients: 83% for illness, 82.9% for severity; caregivers: 79% and 73.7%, respectively). Preferences for information on disease progression, treatment options, side effects, and potential complications were consistently higher among caregivers than patients. Only 38.6% of dialysis patients and 27.6% of non-dialysis patients wanted to know about life expectancy (please see Tables 3a and 3b).

**Table 3a:** Illness-information preferences of ESKD patients on Dialysis and their caregivers *(Frequency of preference for receiving information on illness*,* severity*,* progression*,* treatment options*,* and prognosis)*

Questions	Category	Never No. %	Rarely No. %	Sometimes No. %	Often No. %	Always No. %
A.1.1. Do you prefer to know about your illness?	Patient	16(9.4)	24(14)	75(43.9)	27(15.8)	29(17)
B.1.1. Do you prefer for your patient to know about his/her illness?	Caregiver	10(5.8)	11(6.4)	44(25.7)	38(22.2)	68(39.8)
A.1.2. Do you prefer to know about the severity of your illness?	Patient	35(20.5)	45(26.3)	54(31.6)	13(7.6)	24(14)
B.1.2. Do you prefer for your patient to know about the severity of his/her illness?	Caregiver	23(13.5)	33(19.3)	37(21.6)	25(14.6)	53(31)
A.1.3. Do you prefer to know about the future course of your illness?	Patient	47(27.5)	54(31.6)	39(22.8)	9(5.3)	22(12.9)
B.1.3. Do you prefer your patient to know about the future course of his/her illness?	Caregiver	29(17)	27(15.8)	24(14)	35(20.5)	56(32.7)
A.1.4. Do you prefer to know about your treatment options?	Patient	38(22.2)	36(21.1)	47(27.5)	20(11.7)	30(17.5)
B.1.4. Do you prefer your patient to know about his/her treatment options?	Caregiver	15(8.8)	17(10.0)	32(18.8)	36(21.2)	70(41.2)
A.1.5. Do you prefer to know the success/side-effects of your treatment options?	Patient	39(22.8)	39(22.8)	46(26.9)	16(9.4)	31(18.1)
B.1.5. Do you prefer your patient to know the success/side-effects of his/her treatment options?	Caregiver	16(9.4)	20(11.7)	38(22.2)	44(25.7)	53(31)
A.1.6. Do you prefer to know about the future symptoms?	Patient	49(28.7)	51(29.8)	38(22.2)	8(4.7)	25(14.6)
B.1.6. Do you prefer your patient to know about his/her future symptoms?	Caregiver	28(16.4)	26(15.2)	32(18.7)	37(21.6)	48(28.1)
A.1.7. Do you prefer to know about the future complications due to illness or due to its treatment?	Patient	45(26.3)	39(22.8)	49(28.7)	13(7.6)	25(14.6)
B.1.7. Do you prefer your patient to know about his/her future complications due to illness and its treatment?	Caregiver	23(13.5)	30(17.5)	40(23.4)	34(19.9)	44(25.7)
A.1.8. Do you prefer to know about your expected length of survival?	Patient	105(61.4)	20(11.7)	30(17.5)	0(0)	16(9.4)
B.1.8. Do you prefer your patient to know about his/her expected length of survival?	Caregiver	118(69)	8(4.7)	18(10.5)	9(5.3)	18(10.5)

**Table 3b:** Illness-information preferences of ESKD patients not on Dialysis and their caregivers *(Similar preferences assessed for non-dialysis patients and their caregivers)*

Questions	Category	Never No. %	Rarely No. %	Sometimes No. %	Often No. %	Always No. %
A.1.1. Do you prefer to know about your illness?	Patient	13(17.1)	12(15.8)	23(30.3)	24(31.6)	4(5.3)
B.1.1. Do you prefer for your patient to know about his/her illness?	Caregiver	16(21.1)	13(17.1)	16(21.1)	15(19.7)	16(21.1)
A.1.2. Do you prefer to know about the severity of your illness?	Patient	13(17.1)	18(23.7)	31(40.8)	13(17.1)	1(1.3)
B.1.2. Do you prefer for your patient to know about the severity of his/her illness?	Caregiver	20(26.3)	21(27.6)	12(15.8)	13(17.1)	10(13.2)
A.1.3. Do you prefer to know about the future course of your illness?	Patient	14(18.4)	17(22.4)	29(38.2)	15(19.7)	1(1.3)
B.1.3. Do you prefer your patient to know about the future course of his/her illness?	Caregiver	18(23.7)	21(27.6)	13(17.1)	13(17.1)	11(14.5)
A.1.4. Do you prefer to know about your treatment options?	Patient	13(17.1)	17(22.4)	22(28.9)	18(23.7)	6(7.9)
B.1.4. Do you prefer your patient to know about his/her treatment options?	Caregiver	17(22.4)	17(22.4)	13(17.1)	17(22.4)	12(15.8)
A.1.5. Do you prefer to know the success/side-effects of your treatment options?	Patient	14(18.4)	16(21.1)	23(30.3)	18(23.7)	5(6.6)
B.1.5. Do you prefer your patient to know the success/side-effects of his/her treatment options?	Caregiver	18(23.7)	18(23.7)	13(17.1)	16(21.1)	11(14.5)
A.1.6. Do you prefer to know about the future symptoms?	Patient	14(18.4)	19(25)	24(31.6)	17(22.4)	2(2.6)
B.1.6. Do you prefer your patient to know about his/her future symptoms?	Caregiver	18(23.7)	21(27.6)	13(17.1)	14(18.4)	10(13.2)
A.1.7. Do you prefer to know about the future complications due to illness or due to its treatment?	Patient	15(19.7)	18(23.7)	23(30.3)	17(22.4)	3(3.9)
B.1.7. Do you prefer your patient to know about his/her future complications due To illness and its treatment?	Caregiver	21(27.6)	20(26.3)	11(14.5)	14(18.4)	10(13.2)
A.1.8. Do you prefer to know about your expected length of survival?	Patient	55(72.4)	8(10.5)	5(6.6)	5(6.6)	3(3.9)
B.1.8. Do you prefer your patient to know about his/her expected length of survival?	Caregiver	62(81.6)	2(2.6)	3(3.9)	5(6.6)	4(5.3)

### ACP documentation and decision-making preferences

Although 42.7% of dialysis patients and 69% of their caregivers supported discussing future healthcare preferences, only 20.4% of dialysis patients and 36.3% of caregivers expressed willingness to document ACP. Among non-dialysis patients, 38.1% supported ACP discussions, while 9.2% were willing to document their preferences. Collaborative decision-making was favored by 72.5% of dialysis patients and 85.4% of caregivers, indicating strong family and provider involvement. Among non-dialysis groups, 76.4% of patients and 71% of caregivers preferred shared decision-making. Caregivers consistently endorsed respect for patient preferences more strongly than patients themselves (please see Tables 4a and 4b).

**Table 4a:** Decision-making preferences of ESKD patients on Dialysis and their caregivers *(Levels of agreement on ACP documentation*,* autonomy*,* family/provider roles*,* and shared decision-making)*

Questions	Category	Strongly Disagree No. %	Disagree No. %	Undecided No. %	Agree No. %	Strongly Agree No. %
A.2.1. Future healthcare preferences and options should be discussed and planned	Patient	6(3.5)	42(24.6)	50(29.2)	65(38)	8(4.7)
B.2.1. Future healthcare preferences and options should be discussed and planned	Caregiver	3(1.8)	11(6.4)	39(22.8)	94(55)	24(14)
A.2.2. I prefer my future health care preferences to be documented as an advanced care plan	Patient	6(3.5)	54(31.6)	76(44.4)	30(17.5)	5(2.9)
B.2.2. I prefer my patient’s future healthcare preferences should be documented as an advanced care plan	Caregiver	2(1.2)	18(10.5)	89(52)	49(28.7)	13(7.6)
A.2.3. I prefer to be part of all decision-making discussions concerning my current and future healthcare options	Patient	6(3.5)	27(15.8)	45(26.3)	79(46.2)	14(8.2)
B.2.3. I prefer my patient to be part of all discussions concerning decision-making about his/her current and future healthcare options	Caregiver	0(0)	15(8.8)	29(17)	100(58.5)	27(15.8)
A.2.4. I would prefer to make all the health care decisions myself	Patient	5(2.9)	20(11.7)	34(19.9)	101(59.1)	11(6.4)
B.2.4. I would prefer the patient to make all the health care decisions	Caregiver	2(1.2)	20(11.7)	20(11.7)	102(59.6)	27(15.8)
A.2.5. I would prefer family to make all the health care decisions	Patient	4(2.3)	10(5.8)	25(14.6)	120(70.2)	12(7)
B.2.5. I would prefer the family to make all the health care decisions	Caregiver	0(0)	3(1.8)	16(9.4)	119(69.6)	33(19.3)
A.2.6. I would prefer health care provider (doctors/dialysis team) to make all the health care decisions	Patient	4(2.3)	15(8.8)	27(15.8)	118(69)	7(4.1)
B.2.6. I would prefer health care provider to make all the health care decisions	Caregiver	0(0)	8(4.7)	32(18.7)	118(69)	13(7.6)
A.2.7. I would prefer myself, my family, and healthcare providers (doctors/dialysis team) are involved in the decision-making process	Patient	4(2.3)	12(7)	31(18.1)	112(65.5)	12(7)
B.2.7. I would prefer a shared decision-making process where patient, family, healthcare providers are involved in the decision-making process	Caregiver	1(0.6)	5(2.9)	19(11.1)	118(69)	28(16.4)
A.2.8. I like my preferences, decisions and wishes made about my future treatment and general care process is respected and implemented	Patient	6(3.5)	5(2.9)	75(43.9)	73(42.7)	12(7)
B.2.8. I like my patient’s preferences, decisions, and wishes made about his/ her future treatment, and the general care process is respected and implemented	Caregiver	2(1.2)	1(0.6)	31(18.1)	115(67.3)	22(12.9)

**Table 4b:** Decision-making preferences of ESKD patients not on Dialysis and their caregivers *(Same decision-making themes explored in the non-dialysis cohort)*

Questions	Category	Strongly Disagree No. %	Disagree No. %	Undecided No. %	Agree No. %	Strongly Agree No. %
A.2.1. Future healthcare preferences and options should be discussed and planned	Patient	6(7.9)	15(9.7)	26(34.2)	28(36.8)	1(1.3)
B.2.1. Future healthcare preferences and options should be discussed and planned	Caregiver	0(0)	14(18.4)	25(32.9)	30(39.5)	7(9.2)
A.2.2. I prefer my future health care preferences to be documented as an advanced care plan	Patient	6(7.9)	23(30.3)	40(52.6)	7(9.2)	0(0)
B.2.2. I prefer my patient’s future healthcare preferences should be documented as an advanced care plan	Caregiver	1(1.3)	21(27.6)	37(48.7)	13(17.1)	4(5.3)
A.2.3. I prefer to be part of all decision-making discussions concerning my current and future healthcare options	Patient	4(5.3)	9(11.8)	22(28.9)	33(43.4)	8(10.5)
B.2.3. I prefer my patient to be part of all discussions concerning decision-making about his/her current and future healthcare options	Caregiver	0(0)	13(17.1)	29(38.2)	27(35.5)	7(9.2)
A.2.4. I would prefer to make all the health care decisions myself	Patient	2(2.6)	8(10.5)	13(17.1)	43(56.6)	10(13.2)
B.2.4. I would prefer the patient to make all the health care decisions	Caregiver	0(0)	6(7.9)	23(30.3)	37(48.7)	10(13.2)
A.2.5. I would prefer family to make all the health care decisions	Patient	3(3.9)	6(7.9)	6(7.9)	55(72.4)	6(7.9)
B.2.5. I would prefer the family to make all the health care decisions	Caregiver	0(0)	4(5.3)	13(17.1)	46(60.5)	13(17.1)
A.2.6. I would prefer health care provider (doctors/dialysis team) to make all the health care decisions	Patient	3(3.9)	6(7.9)	12(15.8)	50(65.8)	5(6.6)
B.2.6. I would prefer health care provider to make all the health care decisions	Caregiver	1(1.3)	3(3.9)	15(19.7)	46(60.5)	11(14.5)
A.2.7. I would prefer myself, my family, and healthcare providers (doctors/dialysis team) are involved in the decision-making process	Patient	2(2.6)	6(7.9)	10(13.2)	48(63.2)	10(13.2)
B.2.7. I would prefer a shared decision-making process where patient, family, healthcare providers are involved in the decision-making process	Caregiver	0(0)	4(5.3)	18(23.7)	41(53.9)	13(17.1)
A.2.8. I like my preferences, decisions and wishes made about my future treatment and general care process is respected and implemented	Patient	4(5.3)	8(10.5)	28(36.8)	32(42.1)	4(5.3)
B.2.8. I like my patient’s preferences, decisions and wishes made about his/her future treatment and the general care process is respected and implemented	Caregiver	1(1.3)	5(6.6)	20(26.3)	39(51.3)	11(14.5)

A Chi-square test of independence was conducted to compare the proportions of categorical responses (Yes/No) between dialysis and non-dialysis groups. The results showed a statistically significant difference in the willingness to document Advance Care Planning (ACP) between dialysis and non-dialysis patients (*p* = 0.046). However, the desire to discuss ACP did not show a significant difference between the two groups (*p* = 0.598). Additionally, there were no statistically significant differences found in the willingness to document ACP (*p* = 0.239) or the desire to discuss ACP (*p* = 0.863) among the two caregiver groups.

### Thematic analysis

Qualitative interviews with 40 participants (34 patients and 6 caregivers) yielded three overarching themes: (1) Navigating Uncertainty and Emotional Burden, (2) Reliance on Family and Providers, and (3) Barriers to ACP Engagement. Patients expressed fear about illness progression, financial stress, and trust in physicians. Caregivers described emotional fatigue, lack of preparedness for decisions, and the need for more communication support. Quotes illustrate key themes, e.g., “Dialysis is expensive and tiring” (male, dialysis patient), “We want to help but don’t know what’s best” (female, caregiver). Quotes are drawn from diverse participant types. These findings emphasize that while participants desire information and collaborative involvement, the transition to formal ACP documentation remains hindered by cultural hesitation, limited awareness, and systemic limitations (please see Tables 5a and 5b).

**Table 5a:** Thematic analysis of patient interview responses *(Themes and illustrative quotes from patients on emotional*,* informational*,* financial*,* and Familial experiences)*

Sub-Themes	Participant quotes
Cost of dialysis, loss of income, reliance on the spouse for financial support	“Dialysis weekly requires money,” “My wife has to bear all the income,” “Taking leave for dialysis, my salary cuts.”
Fear of health deterioration, decision-making about treatment, family welfare	“What will be our future?“, “Fear about the decisions,” “Will I get better health after this?”
Spousal support, shared decision-making, reliance on family	“My wife is a big supporter,” “We decide everything together,” “Always being there with me.”
Dietary restrictions, reduced mobility, work-life adjustments	“Living a lifestyle which my doctor said, eating less water,” “Traveling is difficult,” “Illness is very tiring.”
Depression, fear, loss of faith, frustration	“Lost faith in God,” “Can’t believe I got this disease,” “Scared if the treatment goes right.”
Clarifying treatment options, understanding disease progression	“Asking more information about the disease,” “Second opinion about the disease and treatment.”
Work disruptions, fatigue, inability to meet responsibilities	“Taking leave for dialysis,” “Tiredness after dialysis,” “Who will take family responsibilities?”
Exploring alternative therapies, accepting dialysis as the only option	“Should I go for Ayurveda treatment?“, “Dialysis is the only treatment,” “Whatever my doctor says.”
Reliance on family, fear of being a burden	“Who will take care of my family?” “What happens if I can’t make decisions?”

**Table 5b:** Thematic analysis of caregiver interview responses *(Themes and supporting quotes reflecting caregivers’ views on support roles*,* decision burden*,* and communication needs)*

Sub-Themes	Participant quotes
Cost of treatment, insurance concerns	“Money is a big problem spending on dialysis twice a week,” “What can our financial terms be?”
Emotional and practical support, shared decision-making	“I have always been with him for whatever he decided,” “Ready to stop my work and be there with him.”
Fear of health deterioration, planning for the unknown	“What will our kids think?“, “What will be our future since spending so much on treatment?”
Treatment choices, reliance on medical advice	“Choosing the best treatment where the patient can get better,” “Doctor explaining everything about what to expect later.”
Stress, fear, and coping with illness	“Scared what if my decision can cause him anything bad,” “Stress because of what we are going through.”
Fatigue, dietary restrictions, lifestyle limitations	“Dialysis is very tiring and causes cramps,” “Can’t eat all favorite foods, water should be taken less.”
Understanding treatment options and disease progression	“Doctor explained everything,” “Understanding about dialysis till when to continue dialysis.”
Insurance, government support, managing dietary needs	“Government insurance for dialysis,” “Handling medicines and infections.”

We created maps for patient and caregiver responses by hierarchical structuring, with main topics representing questions and subtopics showcasing responses (please refer to supplementary Figs. 1 and 2).

## Discussion

This study highlights two central findings: (1) a pronounced gap exists between the high preference for illness-related information and the low willingness to engage in Advance Care Planning (ACP) documentation, and (2) a strong inclination towards collaborative and family-centered decision-making was expressed by both patients and caregivers. These findings reflect the challenges and opportunities for ACP integration in the Indian ESKD context.

Our results show that while most patients and caregivers desire detailed information about illness and treatment, there is hesitancy when it comes to discussing life expectancy and documenting future care preferences. This aligns with prior evidence from Asian and global studies indicating that, despite a preference for informed care, cultural taboos, fear of distress, and misconceptions about ACP often impede active participation [18–24].

The preference for shared decision-making, especially among dialysis patients and their caregivers, shows the importance of involving families and healthcare providers in ACP processes. However, the reluctance to document preferences suggests that current strategies may not be sufficient to encourage formal ACP engagement. As Adenwalla et al. [25] suggest, behavior-change frameworks that emphasize progressive engagement, rather than immediate action, may be more effective in diverse cultural contexts.

Qualitative findings reveal the emotional, logistical, and informational burdens experienced by patients and caregivers. Financial concerns, emotional fatigue, fear of making incorrect decisions, and dependence on providers emerged as key barriers. These align with global studies that recommend strengthening emotional and informational support systems for both patients and caregivers as a strategy to enhance ACP uptake [26–28].

Our findings also suggest that systemic challenges, such as time constraints, lack of provider training, and absence of structured ACP pathways, must be addressed through institutional interventions. While these were not directly measured, they are consistent with previous studies highlighting operational barriers to ACP conversations in busy nephrology settings [29, 30].

### Limitations

This study has several limitations that warrant consideration. First, the use of a convenience sampling strategy in a single tertiary care hospital in South India may limit the generalizability of the findings to other regions or healthcare settings in India, particularly those with different cultural, linguistic, or socioeconomic contexts. Second, caregiver representation in the qualitative component was limited (*n* = 6), which may have constrained the depth and variability of perspectives regarding barriers to ACP. A larger and more diverse sample could provide richer insights into caregiver experiences. Third, the study relied on self-reported data, which are subject to social desirability and recall biases, especially in discussions surrounding culturally sensitive topics such as death, autonomy, and future care preferences. Additionally, linguistic translation and interpretation of interviews, though carefully conducted, may have inadvertently introduced subtle shifts in meaning, potentially affecting the fidelity of qualitative data. Moreover, while the validated questionnaire captured key domains of ACP engagement, it may not have fully encompassed contextual dimensions, such as the role of religious beliefs or legal awareness, which could influence ACP behaviors. Lastly, the cross-sectional design precludes any assessment of changes in ACP preferences over time or their eventual impact on care outcomes. Longitudinal studies are needed to explore how preferences evolve and whether documented plans are honored in clinical practice.

However, this study adds to the limited literature from India and reinforces that a culturally adapted, inclusive ACP framework is essential. Healthcare providers must be trained to facilitate these conversations sensitively, and institutional protocols should create space and support for ACP discussions to occur regularly and early. Future research should explore longitudinal trajectories of ACP engagement, include healthcare provider perspectives, and test behavior-focused interventions to improve ACP uptake in ESKD and similar chronic illness contexts.

## Conclusion

This study reveals a critical gap between the desire for illness-related information and collaborative decision-making versus the limited engagement in Advance Care Planning (ACP) documentation among ESKD patients and their caregivers in India. Despite expressing high interest in disease-specific knowledge and a preference for family-inclusive decision-making, both patients and caregivers exhibited low readiness to document care preferences. The findings reveal the influence of cultural, emotional, and systemic factors on ACP behavior. Culturally sensitive, behavior-change–driven approaches, including structured communication training for providers and formal inclusion of caregivers in care planning, are vital for enhancing ACP uptake. Addressing these barriers through practical and contextually relevant interventions may ultimately improve patient-centered outcomes in ESKD care. Future research should explore longitudinal trajectories of ACP engagement, incorporate diverse cultural perspectives, and examine interventions that support progressive patient and caregiver readiness for ACP documentation.

### Practice implication

Our findings highlight several key areas to inform improvements in Advance Care Planning (ACP) practice for patients with End-Stage Kidney Disease (ESKD) in India. First, ACP discussions must be normalized and integrated into standard nephrology care pathways early in the course of illness. These conversations should be conducted by healthcare providers who are trained in culturally sensitive communication and supported by institutional protocols. Second, caregiver involvement should be explicitly included as part of the ACP process. Resources such as informational materials, caregiver support groups, and facilitated dialogue sessions can equip families to participate more meaningfully in shared decision-making. Third, behavior-change approaches—such as repeated low-stakes discussions rather than one-time ACP documentation—can help patients and caregivers gradually engage in planning without feeling overwhelmed or culturally conflicted. Finally, systemic efforts such as allocating time for ACP in clinical schedules, embedding ACP prompts in electronic medical records, and enabling provider education initiatives are essential to bridge the gap between patient preferences and clinical realities. These strategies, grounded in the local context, may foster patient- and family-centered ACP engagement and improve care alignment with patient values. of ACP engagement, incorporate diverse cultural perspectives, and examine interventions that support progressive patient and caregiver readiness for ACP documentation.

## Supplementary Information

Below is the link to the electronic supplementary material.

Supplementary Material 1

Supplementary Material 2

## Data Availability

Data is provided within the manuscript or supplementary information files.

## Abbreviations

ACP: Advance Care Planning
CKD: Chronic Kidney Disease
ESKD: End-Stage Kidney Disease
eGFR: Estimated Glomerular Filtration Rate
MAHE: Manipal Academy of Higher Education
SPSS: Statistical Package for the Social Sciences
