# Supplementary figures and images for: Barriers and preferences in advance care planning: a mixed-methods study of end-stage kidney disease patients and caregivers in India
Source: BMC Nephrol. 2025 Jul 24;26:415. doi: 10.1186/s12882-025-04354-2 (PMC12291236; doi:10.1186/s12882-025-04354-2)

**Supplementary Figure 1. Mind map of patient responses from qualitative interview**


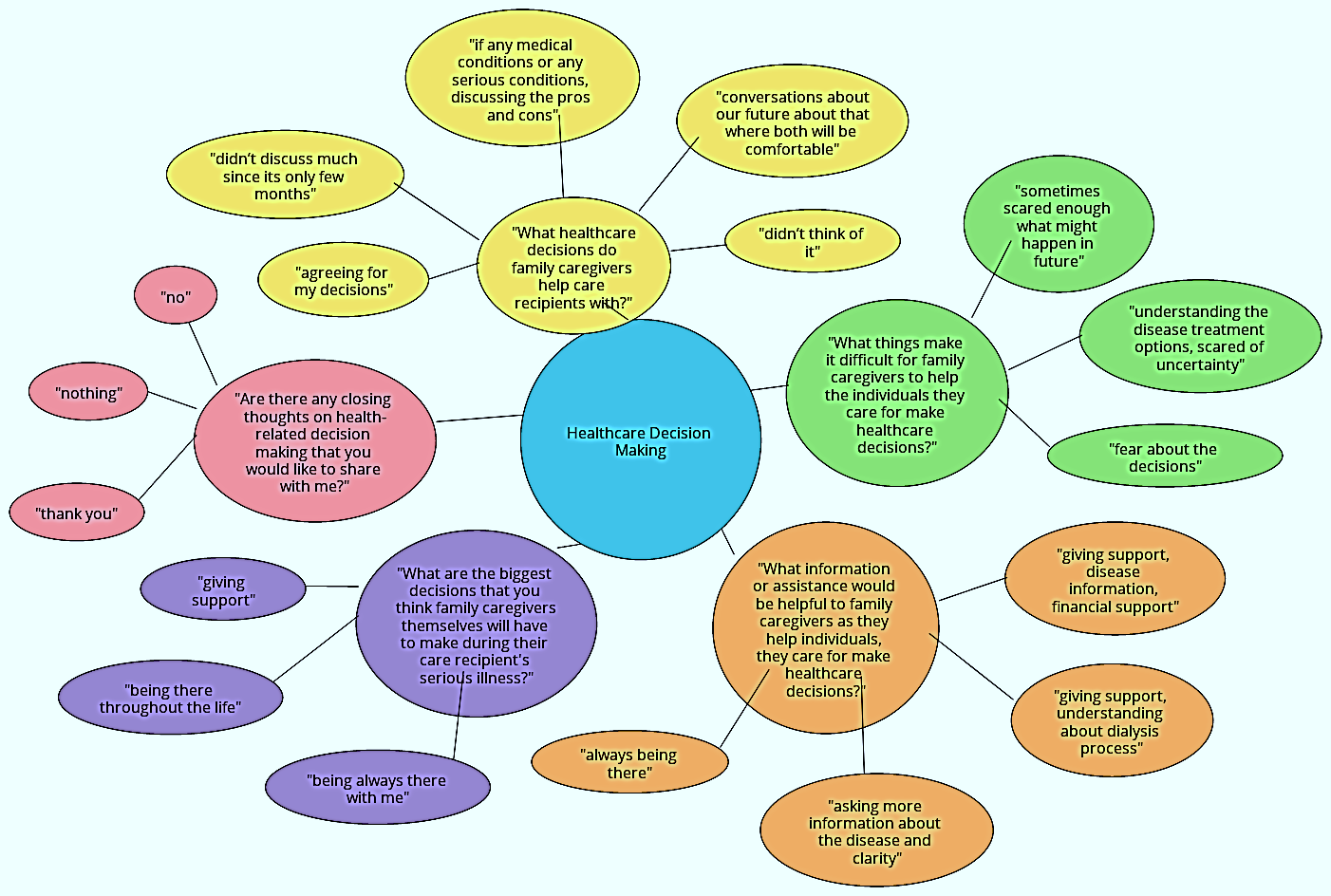

Supplement: Supplementary file 1 — Supplementary Material 1 [file 12882_2025_4354_MOESM1_ESM.docx]

**Supplementary Figure 2. Mind map of caregiver responses from qualitative interview**


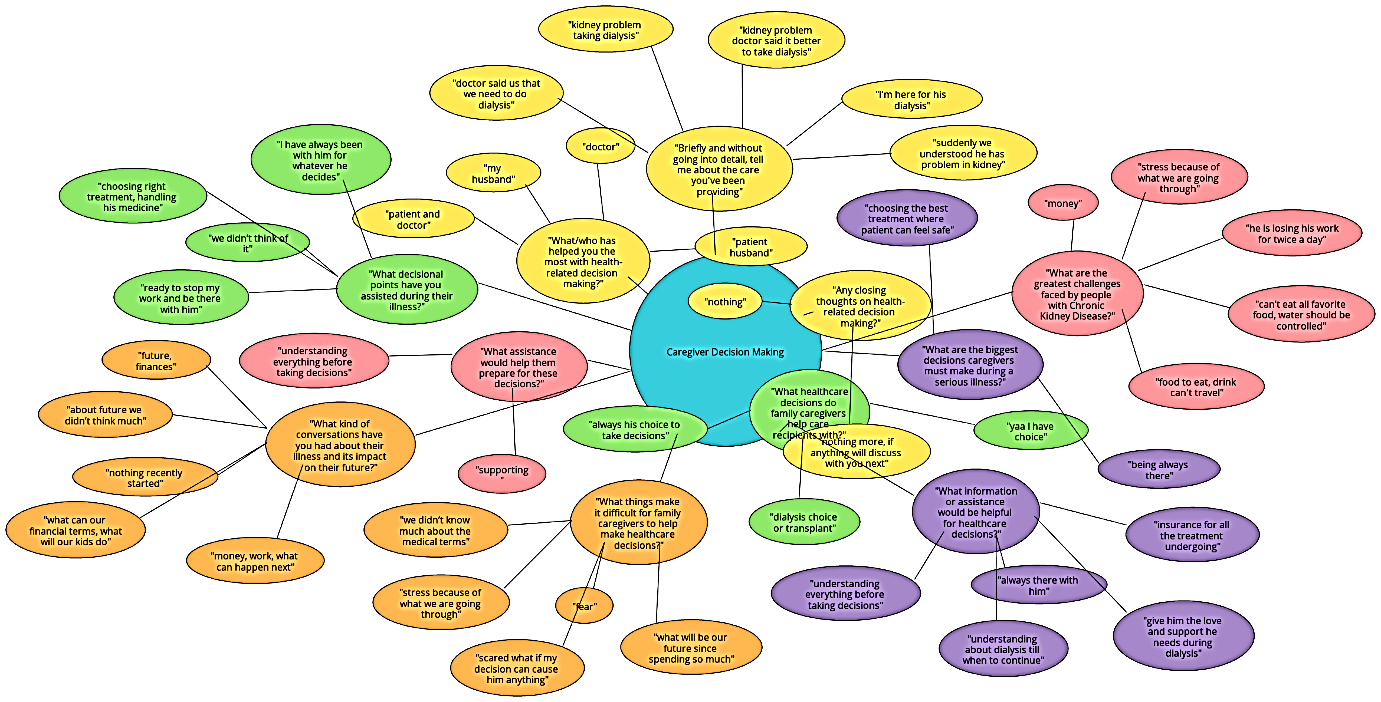

Supplement: Supplementary file 2 — Supplementary Material 2 [file 12882_2025_4354_MOESM2_ESM.docx]
